# Elevational Variation in Morphology and Biomass Allocation of Alpine Yellow-Violet *Viola biflora*

**DOI:** 10.3390/plants15213368

**Published:** 2026-10-31

**Authors:** Piotr Kiełtyk, Julia Piwowarska, Piotr Dąbrowski

**Affiliations:** 1 Institute of Biological Sciences, Cardinal Stefan Wyszynski University in Warsaw, Wóycickiego 1/3, 01-938 Warsaw, Poland; 2 Faculty of Biology and Environmental Sciences, Cardinal Stefan Wyszynski University in Warsaw, Wóycickiego 1/3, 01-938 Warsaw, Poland; juliapiwowarska29@gmail.com; 3 Department of Environmental Development and Remote Sensing, Faculty of Civil Engineering, Environmental Engineering and Architecture, Warsaw University of Life Sciences, Nowoursynowska 159, 02-776 Warsaw, Poland; piotr_dabrowski@sggw.edu.pl

**Keywords:** elevational gradient, forest–alpine transition, leaf morphology, phenotypic variation, reproductive allocation, trait variation

## Abstract

Mountain plants spanning broad elevational ranges experience changes in climate, vegetation structure and biotic interactions that may generate contrasting trait responses. We investigated elevational variation in morphology and aboveground biomass allocation of *Viola biflora* across its range in the Polish Tatra Mountains. A total of 338 flowering individuals were sampled at 34 sites between 890 and 2115 m a.s.l. Traits were analysed using mixed-effects models comparing straight-line and quadratic relationships with elevation. Plant height, plant mass, stem mass and rosette and stem leaf petiole length showed hump-shaped relationships, with maxima around the middle of the elevational range. Plant height increased by 22.9% to its maximum and then decreased by 34.1%, while stem leaf petiole length increased by 75.6% before declining by 34.5%. Leaf mass and stem leaf blade area decreased continuously with elevation. Flower mass did not vary significantly, whereas corolla length and width decreased by 15.5% and 19.6%, respectively. Flower mass fraction was lowest at intermediate elevations and increased towards the highest sites. Mid-elevation maxima in plant height and petiole length coincided with the transition from forest understorey to open subalpine vegetation and may reflect changes in light availability and interactions with neighbouring vegetation.

## 1. Introduction

Mountain slopes expose plant populations to pronounced environmental variation over relatively short geographical distances. In temperate mountains, increasing elevation is generally accompanied by declining air temperature and a shorter growing season, as well as changes in solar radiation, wind exposure, snow duration, soil development and nutrient availability [1,2,3]. Biotic conditions also change with elevation. Competition among plants and herbivory generally decline towards higher elevations [2,4], whereas limitations associated with pollinator availability may become more pronounced in colder high-elevation environments [5,6,7]. Elevation therefore represents a composite environmental gradient integrating numerous abiotic and biotic factors whose relative importance may differ along a mountain slope. Plant populations may respond to this environmental heterogeneity through phenotypic plasticity, genetically based differentiation among populations, or a combination of both processes [8,9,10,11,12,13,14,15,16,17,18]. Such responses may be expressed as changes in plant stature, organ dimensions and biomass investment in vegetative and reproductive structures. Reductions in plant height and leaf area are commonly observed towards higher elevations, although their magnitude and direction vary among species and growth forms [19,20]. Changes in biomass allocation among leaves, stems and reproductive structures may additionally reflect differences in resource investment associated with acquisition, competitive ability, growth and reproduction [21,22,23].

The general tendency towards reduced plant stature, together with variation among species, is illustrated by the findings of Rixen et al. [20], who examined 66 alpine plant species from Switzerland, China, Australia and New Zealand. They found that plant stature generally decreased with elevation, although the magnitude of trait variation depended on species’ elevational preferences and ranges. In the Swiss Alps, higher-elevation populations of *Poa alpina* L. exhibited reduced growth and relative reproductive investment [24], whereas higher-elevation populations of *Saxifraga trifurcata* Schrad. in northern Spain showed greater vegetative growth per unit canopy area but lower reproductive investment [25]. In central Japan, plants of the *Solidago virgaurea* L. complex growing at higher elevations had shorter stems but larger flower heads [26], while floral dimensions decreased with elevation in *Impatiens textorii* Miq. but not in *I. noli-tangere* L. [27]. More recently, increased leaf dimensions and flower width towards higher elevations were documented in Himalayan *Thymus linearis* Benth. [28]. In the Apennine Mountains, Amoroso et al. [29] reported a general decline in leaf area with elevation, whereas flower size often increased, particularly in single-flowered species with broad elevational ranges. Together, these findings illustrate that vegetative and reproductive traits may exhibit contrasting elevational responses, and that these patterns are species- and trait-specific.

Trait variation along elevational gradients, however, need not follow simple monotonic trends (e.g., [30]). Although temperature generally decreases progressively with elevation, other environmental factors, including vegetation structure, light availability, soil properties and biotic interactions, can change in different ways across a mountain slope. Consequently, different environmental pressures may predominate in different parts of a species’ elevational range and generate curvilinear or threshold responses [31]. Curvilinear relationships between plant morphological traits and elevation have been documented in several mountain species. In the Tatra Mountains, for example, numerous floral traits of *Solidago minuta* L. showed unimodal relationships with elevation, with their maxima occurring near the centre of the species’ elevational distribution [32]. Such patterns may remain undetected when only a few elevation levels are sampled, highlighting the value of dense sampling across a species’ full vertical range when testing the shape of trait–elevation relationships [33].

Studies conducted along broad elevational gradients in the Tatra Mountains have revealed considerable differences among species in morphological and biomass-allocation responses. In the wide-ranging *Bellidiastrum michelii* Cass., plant height, aboveground mass and leaf mass decreased markedly with elevation, while changes in floral traits were generally less pronounced [34]. *Senecio subalpinus* W.D.J.Koch likewise showed decreasing height and aboveground biomass, but reproductive investment changed differently, including an increase in individual flower mass towards higher elevations [35]. In *Soldanella carpatica* Vierh., leaf mass, leaf size and scape height decreased through most of the gradient, whereas flower mass remained comparatively stable and flower mass fraction increased with elevation [36]. More recently, a comparative analysis showed that plant height decreased with elevation in all five examined Tatra species, whereas the high-elevation specialists *Campanula alpina* Jacq. and *Doronicum stiriacum* (Vill.) Dalla Torre maintained considerably more stable aboveground biomass than species with wider or lower elevational distributions [33]. These contrasting responses indicate that even species occurring within the same mountain system may differ substantially in how their morphology and biomass allocation change across elevation.

*Viola biflora* L. (Violaceae) is a perennial herb characterised by pale-yellow flowers, occurring in cool, moist habitats from montane forests to alpine vegetation [37,38,39,40]. Its broad elevational distribution in the Tatra Mountains makes it particularly suitable for investigating intraspecific morphological variation and biomass allocation across contrasting vegetation environments. At lower elevations, the species occurs mainly in forest understorey, with occasional occurrence at forest edges, whereas towards the upper part of its range it occupies open subalpine and alpine vegetation. Thus, populations experience not only a pronounced climatic gradient, but also substantial changes in vegetation structure and light environment across their elevational distribution. In the present study, we sampled *V. biflora* across 34 sites along an elevational gradient spanning 1225 m to examine how vegetative morphology, floral traits and aboveground biomass allocation vary across this environmental transition. Specifically, we asked (1) whether vegetative and floral traits and biomass-allocation patterns of *V. biflora* vary significantly with elevation, and (2) whether these responses follow monotonic trends or instead show curvilinear patterns along the elevational gradient. We hypothesised that (H1) plant stature and vegetative organ dimensions would decrease towards the upper part of the species’ elevational range, reflecting increasingly restrictive climatic conditions at high elevations. However, because *V. biflora* also crosses a major transition from forest to open vegetation, we further hypothesised that (H2) traits associated with vertical leaf positioning, particularly plant height and petiole length, might exhibit non-monotonic relationships with elevation.

## 2. Results

**Figure 1:**
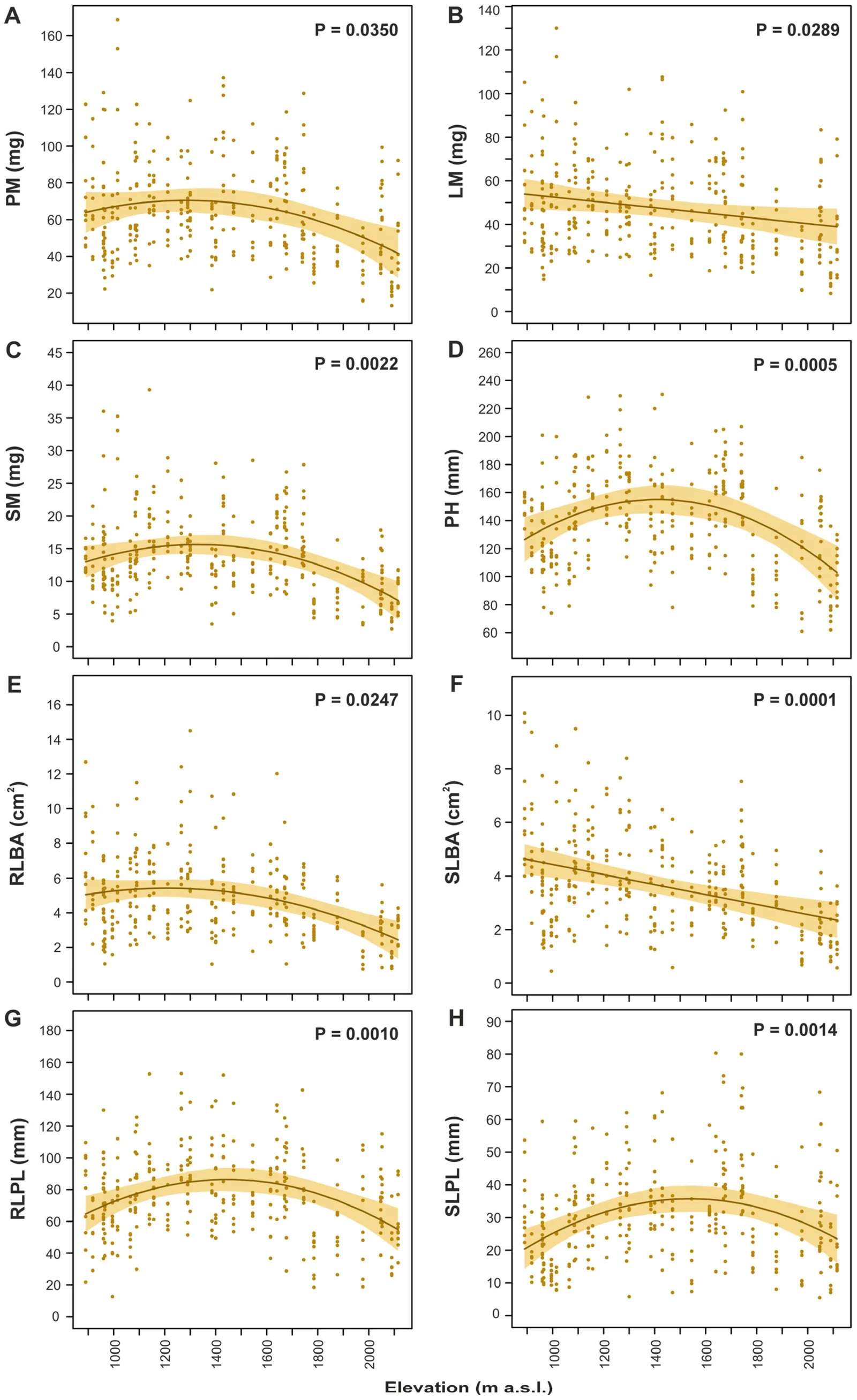
Elevational variation in vegetative traits of *Viola biflora*. (**A**) Plant mass (PM), (**B**) leaf mass (LM), (**C**) stem mass (SM), (**D**) plant height (PH), (**E**) rosette leaf blade area (RLBA), (**F**) stem leaf blade area (SLBA), (**G**) rosette leaf petiole length (RLPL), (**H**) stem leaf petiole length (SLPL). Solid lines represent fitted relationships from the selected mixed-effects models, and shaded bands indicate 95% confidence intervals. *P* denotes the *p*-value for the elevation term of the selected model (linear term for straight-line models and quadratic term for quadratic models).

All vegetative traits varied significantly along the 890–2115 m a.s.l. elevational gradient (Figure 1; Table 1 and Table 2). Plant mass and stem mass showed hump-shaped relationships with elevation (*P* = 0.0350 and 0.0022, respectively). Plant mass increased by 10.7% from the lowest elevation to a maximum at approximately 1290 m a.s.l. and then decreased by 41.6% towards the highest elevation. Stem mass increased by 20.8% to a maximum at approximately 1330 m a.s.l. and subsequently decreased by 55.1%.

Plant height and both petiole-length traits showed pronounced hump-shaped relationships with elevation (Figure 1D,G,H). Their maxima occurred within a relatively narrow elevational range, between approximately 1410 and 1540 m a.s.l. Plant height increased by 22.9% from 890 m a.s.l. to a maximum at approximately 1410 m a.s.l. and then decreased by 34.1% towards 2115 m a.s.l. (*P* = 0.0005). Rosette leaf petiole length increased by 31.2% to a maximum at approximately 1440 m a.s.l. and subsequently decreased by 36.4% (*P* = 0.0010). Stem leaf petiole length showed the strongest response, increasing by 75.6% to a maximum at approximately 1540 m a.s.l. before decreasing by 34.5% towards the highest elevation (*P* = 0.0014; Table 1).

In contrast, leaf size and leaf biomass generally declined towards higher elevations (Figure 1B,E,F). Rosette leaf blade area showed only a slight initial increase of 7.7% to a maximum at approximately 1215 m a.s.l., followed by a 54.9% decrease towards the highest elevation (*P* = 0.0247). Stem leaf blade area declined continuously with elevation by 49.2% (*P* = 0.0001), while leaf mass decreased by 28.2% across the gradient (*P* = 0.0289; Table 1 and Table 2).

**Figure 2:**
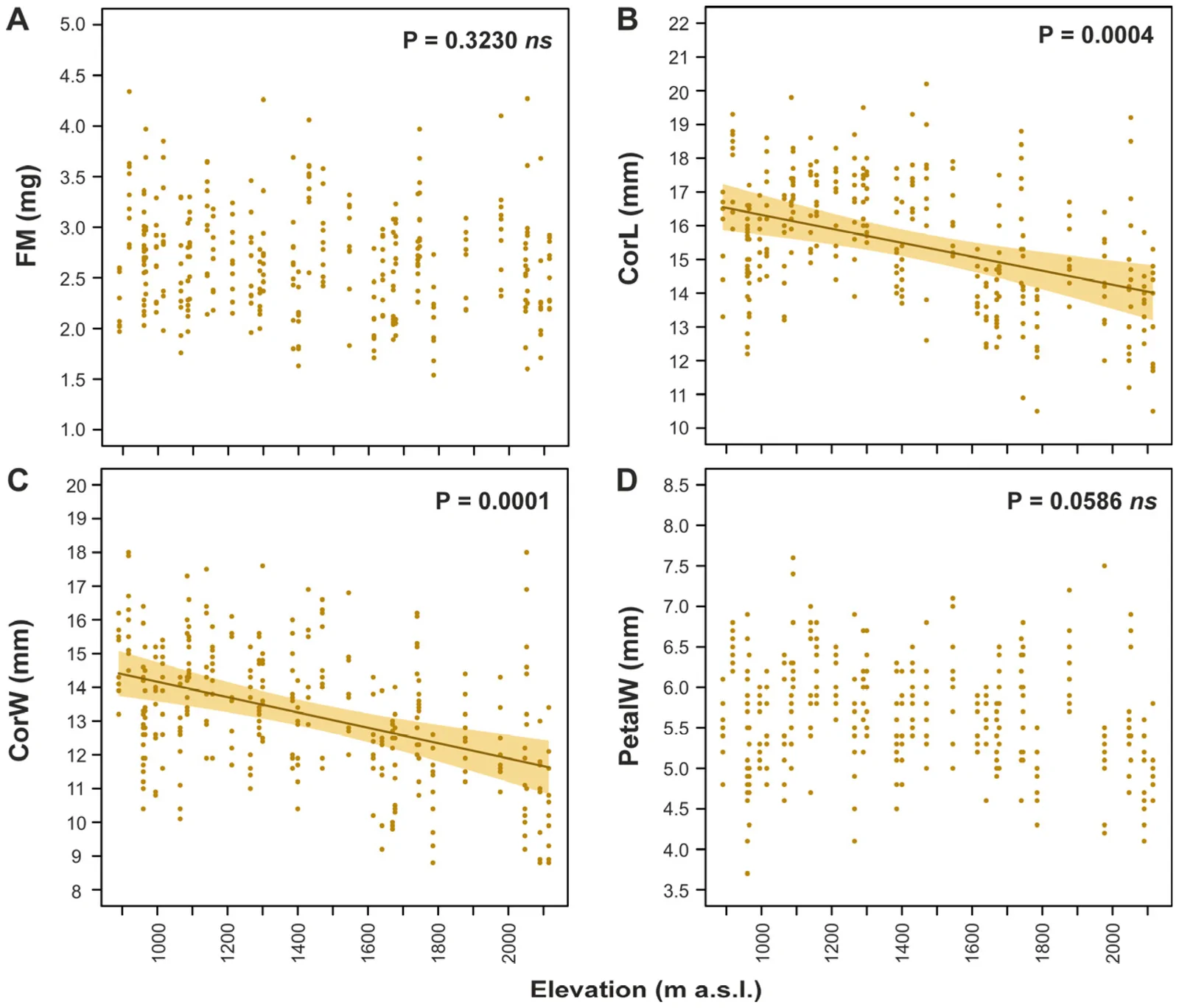
Elevational variation in floral traits of *Viola biflora*. (**A**) Flower mass (FM), (**B**) corolla length (CorL), (**C**) corolla width (CorW), (**D**) lower petal width (PetalW). For significant relationships, solid lines represent fitted relationships from the selected mixed-effects models, and shaded bands indicate 95% confidence intervals. *P* denotes the *p*-value for the elevation term of the selected model (linear term for straight-line models and quadratic term for quadratic models); *ns*, non-significant at *P* ≥ 0.05.

Flower mass (*P* = 0.3230) and petal width (*P* = 0.0586) did not vary significantly with elevation (Figure 2A,D). In contrast, corolla length and corolla width decreased significantly along the gradient, declining by 15.5% (*P* = 0.0004) and 19.6% (*P* = 0.0001), respectively, from the lowest to the highest elevation (Figure 2B,C; Table 1 and Table 2).

**Figure 3:**
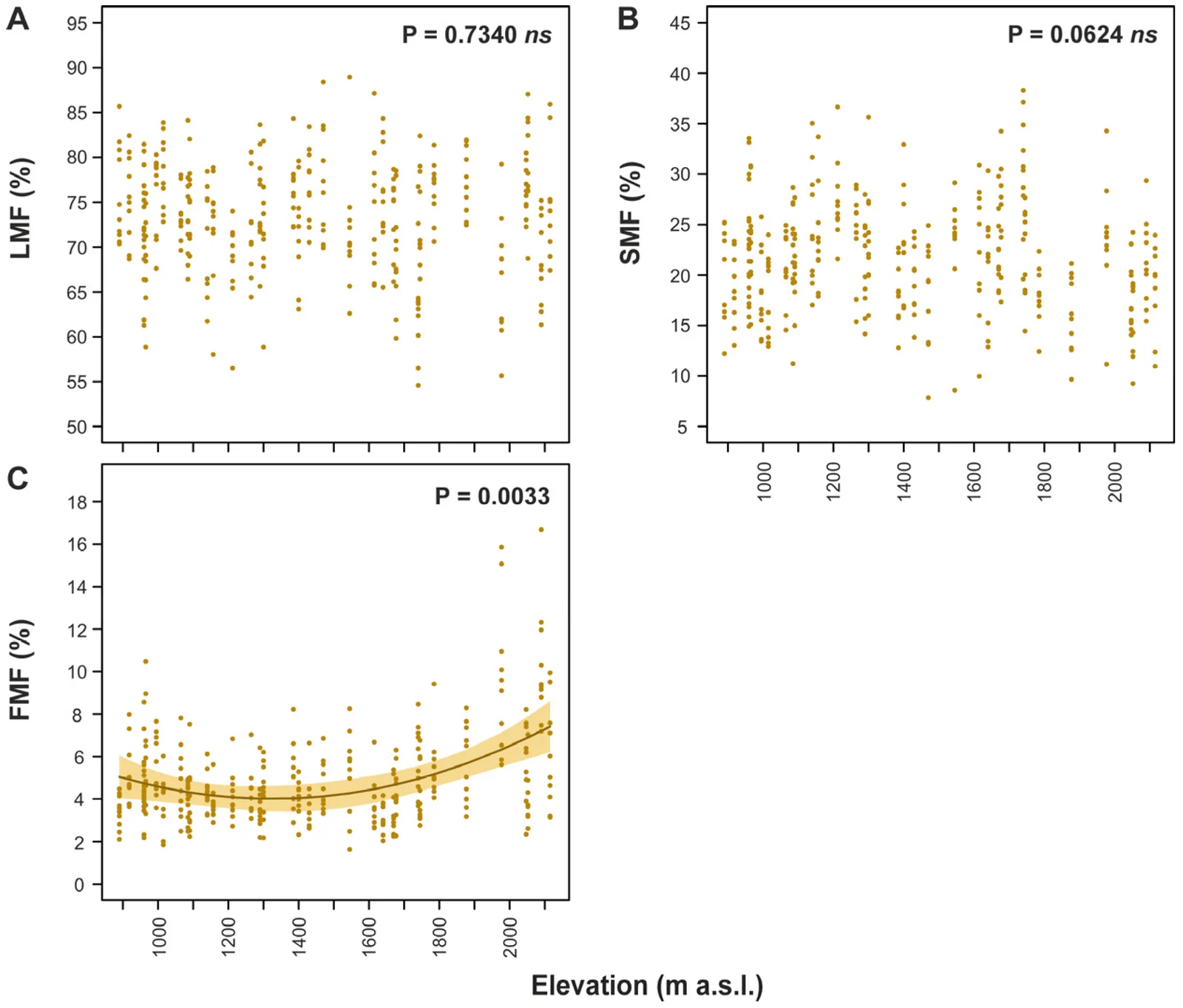
Elevational variation in biomass-allocation traits of *Viola biflora*. (**A**) Leaf mass fraction (LMF), (**B**) stem mass fraction (SMF), (**C**) flower mass fraction (FMF). For significant relationships, solid lines represent fitted relationships from the selected mixed-effects models, and shaded bands indicate 95% confidence intervals. *P* denotes the *p*-value for the elevation term of the selected model (linear term for straight-line models and quadratic term for quadratic models); *ns*, non-significant at *P* ≥ 0.05.

Leaf mass fraction (*P* = 0.7340) and stem mass fraction (*P* = 0.0624) showed no significant relationship with elevation (Figure 3A,B). In contrast, flower mass fraction varied significantly with elevation (*P* = 0.0033), decreasing by 20.7% from the lowest elevation to a minimum at approximately 1330 m a.s.l. and subsequently increasing by 85.8% towards the highest elevation (Figure 3C; Table 1). Its fitted value at 2115 m a.s.l. was therefore 47.3% higher than at 890 m a.s.l.

## 3. Discussion

*Viola biflora* showed pronounced but contrasting morphological responses along an elevational gradient spanning 1225 m. The clearest pattern was the hump-shaped variation in plant height and both petiole-length traits, which increased from the lower part of the gradient, reached maxima around the middle of the species’ elevational range, and then declined towards the highest elevations. Plant and stem mass showed similar unimodal responses, whereas leaf mass and stem leaf blade area decreased continuously with elevation and rosette leaf blade area showed only a weak initial increase before declining markedly. Thus, different components of plant morphology did not respond uniformly to elevation. Similar trait-specific responses have been reported for *Gentiana lawrencei* Burkill in alpine grasslands, where leaf size exhibited a hump-shaped relationship with elevation, whereas aboveground plant mass declined linearly [30]. Importantly, the mid-elevation maxima in plant height and petiole length in *V. biflora* broadly coincided with the transition from predominantly forest understorey habitats to more open subalpine vegetation. This correspondence suggests that morphological variation in *V. biflora* may reflect not only increasingly restrictive climatic conditions with elevation, but also changes in vegetation structure, light environment and interactions with neighbouring plants.

The pronounced hump-shaped responses of plant height and petiole length in *V. biflora* are particularly relevant because these traits influence the vertical positioning of leaves within the surrounding vegetation. Stem and petiole elongation can improve access to light under shading and are important components of shade-avoidance responses [41,42,43]. At lower elevations, *V. biflora* occurred predominantly beneath forest canopies, where limited light availability may constrain the growth of both *V. biflora* and neighbouring herbaceous vegetation [44,45]. Around the middle of the elevational range, where *V. biflora* increasingly occurred outside closed forest, greater light availability combined with relatively favourable thermal conditions may have promoted more vigorous vegetation growth and increased competition for light. Zhu et al. [46] also found that the proportion of plants exhibiting a competitive (C) strategy peaked at intermediate elevations along a broad mountain elevational gradient, suggesting that competition may become particularly important under such conditions. The maxima in plant height and petiole length in *V. biflora* may therefore reflect enhanced growth under relatively favourable environmental conditions, potentially accompanied by greater benefits of positioning leaves above neighbouring vegetation. However, competition is not necessarily the principal driver of elevational variation in plant height. In a study of *Dianthus carthusianorum* L. in the Swiss Alps, evolutionary modelling suggested that abiotic conditions were more important than interspecific competition in driving elevational differentiation in plant height, although competition increased relative investment in height growth [47]. These findings highlight the potentially different contributions of abiotic conditions and competition to variation in vertical growth. Because light availability, surrounding vegetation structure and microclimatic conditions were not measured directly in the present study, their relative contributions to the observed patterns in *V. biflora* cannot be determined.

Above their mid-elevation maxima, plant height and both petiole lengths declined markedly towards the upper limit of the species’ range. This shift is consistent with the increasing importance of abiotic constraints at high elevations, where low temperature, short growing seasons, strong winds and limited resource availability restrict plant growth [2]. More generally, the relative importance of competitive interactions tends to decrease as environmental stress increases towards high elevations [48]. Declining plant height with elevation is among the most consistent morphological responses reported for mountain plants [19,20], including several species studied in the Tatra Mountains [32,33,34,35,36]. Smaller stature at high elevations may result directly from restricted growth, but it may also be advantageous because plant tissues remain closer to the ground, where temperatures are often higher and wind speeds lower than in the free atmosphere [2]. The decline in stature of *V. biflora* towards its upper elevational limit is therefore consistent with the general shift from conditions permitting greater vertical growth at intermediate elevations to increasingly restrictive alpine conditions.

Leaf blade traits showed a different elevational pattern. Stem leaf blade area declined continuously, while rosette leaf blade area showed only a small increase at the lower part of the gradient before decreasing strongly towards high elevations. Leaf mass likewise declined progressively with elevation. These responses contrast with the pronounced mid-elevation maxima in plant height and petiole length and indicate that increased plant stature at intermediate elevations was not accompanied by a corresponding increase in leaf biomass or leaf blade area. Reductions in leaf size towards higher elevations are common in mountain plants and have been associated with increasingly cold and resource-limited environments [19,49,50]. A general tendency for leaf area to decline with increasing elevation was also reported in the intraspecific analyses of a multispecies study from the Apennine Mountains [29], supporting the view that reduced leaf dimensions can be a common, although not universal, response within species to high-elevation environments. Similarly, Sharma et al. [51] reported decreases in leaf area and leaf dry mass with increasing elevation in *Parthenium hysterophorus* L. in the western Himalayas, although several other leaf traits exhibited curvilinear relationships. Conversely, Wani et al. [28] found that several leaf dimensions increased towards higher elevations in Himalayan *Thymus linearis*. These contrasting findings further demonstrate that reductions in leaf size are not a universal response to increasing elevation. In *V. biflora*, comparatively larger leaf blades at lower elevations may also be advantageous under shaded forest conditions because greater surface area can enhance light interception. The observed decline in both leaf mass and blade area towards the upper part of the gradient therefore appears consistent with a shift from relatively greater investment in photosynthetic surface at lower elevations to smaller leaves under increasingly severe high-elevation conditions. Such reductions in leaf size may represent an advantageous adjustment to low temperatures, strong radiation, wind exposure and associated constraints on water and energy balance [52,53].

Floral traits of *V. biflora* responded less uniformly than vegetative traits. Flower dry mass did not vary significantly with elevation, and petal width was also relatively stable, whereas corolla length and width declined by approximately 16–20% across the gradient. Thus, the amount of dry biomass invested in the flower showed no significant elevational trend even though some dimensions of the corolla decreased. Such trait-specific responses are consistent with recent evidence from Himalayan mountain plants, where intraspecific variation in floral traits showed highly species-specific elevational patterns [54]. Similarly, in central Japan, floral dimensions decreased with elevation in *Impatiens textorii* but showed no significant elevational trends in *I. noli-tangere* [27]. In contrast, flower width increased towards higher elevations in Himalayan *Thymus linearis* [28]. Interestingly, Amoroso et al. [29] reported a general tendency for flower size to increase with elevation in plants from the Apennine Mountains, although the direction and strength of the response varied among species. Increases were particularly evident in single-flowered species occupying broad elevational ranges. The decline in corolla dimensions observed in *V. biflora* further illustrates that floral-size responses to elevation can differ markedly among species. The absence of a detectable elevational trend in flower mass of *V. biflora* may nevertheless indicate that biomass investment in the flower was relatively conserved compared with vegetative growth. Similar stability of individual flower mass has been reported for some other Tatra mountain species, particularly *Campanula alpina* and *Doronicum stiriacum*, whereas stronger decreases occurred in species such as *Soldanella carpatica* and *Bellidiastrum michelii* [33]. Such interspecific differences suggest that reproductive traits may be less uniformly responsive to elevation than vegetative size traits.

The elevational pattern in flower mass fraction provides additional insight into this contrast between vegetative and floral biomass. Flower mass fraction decreased by 20.7% from the lowest elevation to a minimum at approximately 1330 m a.s.l. and then increased by 85.8% towards the highest elevation. Notably, this minimum occurred almost exactly within the elevational range at which plant and stem mass reached their maxima. Because flower mass itself did not change significantly, the reduction in flower mass fraction at intermediate elevations appears to result primarily from increased investment in vegetative biomass rather than from reduced absolute investment in flowers. Conversely, the strong increase in flower mass fraction towards the upper part of the gradient largely reflects the decline in vegetative biomass while flower mass remains comparatively stable. The higher flower mass fraction at high elevations therefore reflects a greater proportional contribution of flowers to aboveground biomass, rather than an increase in absolute flower biomass. Similar increases in relative reproductive allocation with elevation have been reported for other Tatra species, including *Bellidiastrum michelii*, *Campanula alpina*, *Senecio subalpinus* and *Soldanella carpatica* [33,34,35,36].

Reduced stature at high elevations may additionally influence the microclimatic conditions experienced by flowers. Because near-ground temperatures can substantially exceed free-air temperatures under suitable radiation conditions, shorter reproductive shoots may maintain flowers closer to the relatively warm boundary layer [2]. Floral temperature may affect pollinator behaviour and reproductive processes, particularly under cool mountain conditions [55,56,57]. The combination of reduced plant stature and maintained flower biomass in high-elevation *V. biflora* may therefore contribute to maintaining reproductive function under increasingly cold conditions. In addition, *V. biflora* is capable of cleistogamous self-pollination [38], which may provide an alternative route to reproduction when conditions for pollinator activity or successful cross-pollination are less favourable. The relative importance of chasmogamous and cleistogamous reproduction may therefore also contribute to reproductive persistence across the species’ broad elevational range.

The present study demonstrates pronounced and trait-specific phenotypic variation in *V. biflora* across a broad elevational gradient, including distinctive hump-shaped responses of plant height and petiole length whose maxima broadly coincided with the transition from forest understorey to more open subalpine vegetation. This correspondence is consistent with morphological variation across the species’ elevational range being associated not only with progressively changing climatic conditions, but also with major changes in the vegetation environment. Nevertheless, elevation integrates numerous environmental factors, including temperature, snow duration, soil properties, moisture availability, air humidity, slope exposure, wind and interactions with neighbouring vegetation, and their individual contributions cannot be separated from the present observational data. Moreover, light availability, temperature and humidity can vary among sites and throughout the day, and these microclimatic differences were not quantified. In particular, because light conditions and surrounding vegetation structure were not quantified, the proposed role of changing light availability and competition in producing the observed mid-elevation maxima cannot be assessed directly. Measurements of light quantity and quality, vegetation structure and microclimatic conditions along the gradient would allow these mechanisms to be tested more explicitly. Likewise, the present field data do not distinguish phenotypic plasticity from genetically based differentiation among populations. Common-garden experiments and reciprocal transplantations would therefore provide an important next step for determining the relative contributions of environmental responses and persistent population differentiation to the observed elevational patterns [8,11,13,14].

## 4. Materials and Methods

### 4.1. Study Area

**Figure 4:**
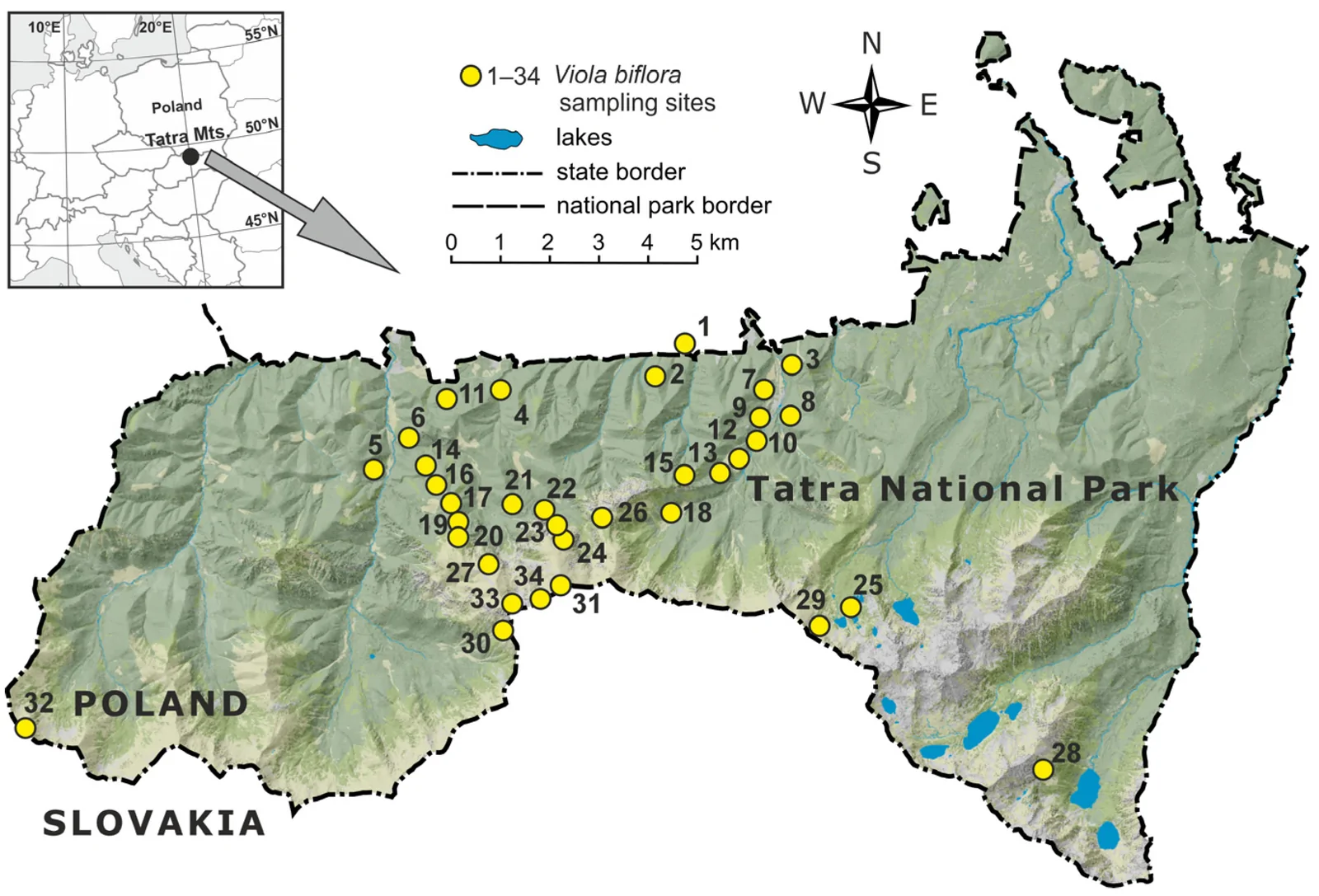
Location of the 34 sampling sites of *Viola biflora* in the Polish Tatra Mountains, Western Carpathians. Numbered circles correspond to sampling sites listed in Table 3. The inset shows the location of the Tatra Mountains in Europe.

The study was conducted in the Polish Tatra Mountains, Western Carpathians, within Tatra National Park (Figure 4, Table 3). The Tatras are the highest mountain range in the Carpathians, reaching 2655 m a.s.l. at Gerlachovský štít in Slovakia. In the Polish part of the range, elevations extend from approximately 900 m a.s.l. to 2499 m a.s.l. at Rysy, and the landscape is dominated by mountain valleys and slopes with predominantly northern exposure.

Climatic conditions change markedly with elevation. Mean annual air temperature declines from approximately +6 °C in the foothills to about −4 °C at the highest summits, while the duration of snow cover increases from approximately 100 days at lower elevations to nearly 290 days near the summits [58]. This climatic gradient is accompanied by a pronounced zonation of vegetation. Five major elevational belts are distinguished in the Polish Tatras: the lower montane forest belt, extending to approximately 1200 m a.s.l.; the upper montane forest belt, from 1200 to 1550 m a.s.l.; the subalpine belt dominated by dwarf pine, from 1550 to 1800 m a.s.l.; the alpine belt, from 1800 to 2250 m a.s.l.; and the subnival belt above approximately 2250 m a.s.l. [59].

### 4.2. Study Species

**Figure 5:**
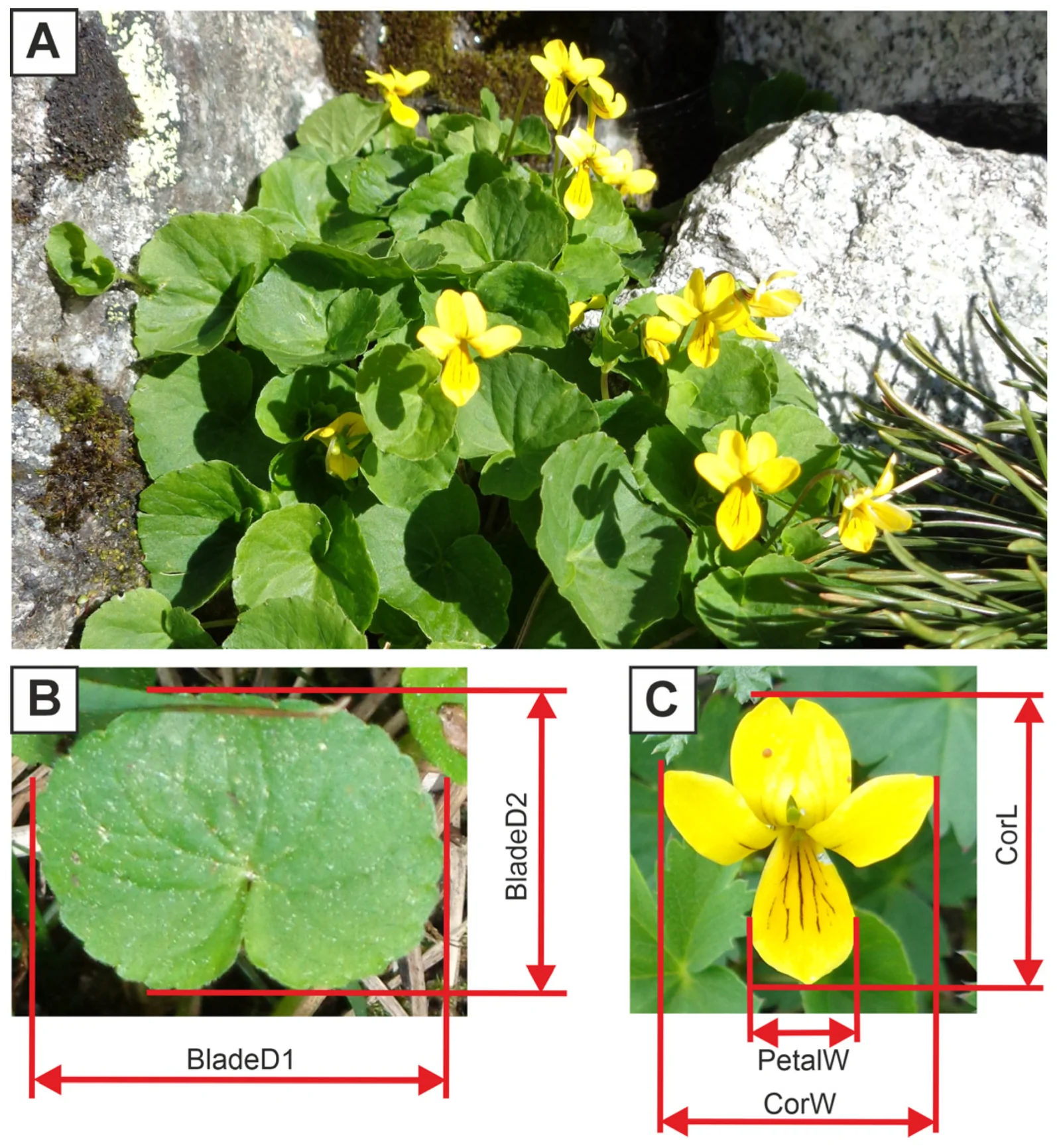
*Viola biflora* in its natural habitat (**A**), and measurements of leaf blade (**B**) and floral traits (**C**). BladeD1, maximum leaf blade diameter; BladeD2, leaf blade diameter perpendicular to BladeD1; CorL, corolla length; CorW, corolla width; PetalW, lower petal width.

*Viola biflora* (Figure 5A) has a slender ascending stem typically 5–20 cm tall and bears two to four leaves and one to two flowers [37]. The leaves are cordate to reniform, with crenate margins, thin blades and slight pubescence along the veins on their lower surface. Flowers are borne on pedicels and have a pale-yellow corolla marked with brownish veins. The corolla consists of five petals, with the lower petal bearing a short spur approximately 2–3 mm long. The fruit is a glabrous, three-valved capsule, and the seeds bear an elaiosome. The species flowers mainly from May to July and fruits from July to October. Reproduction involves insect-pollinated flowers as well as cleistogamous self-pollination [38,39,40].

*Viola biflora* occurs in forest understorey vegetation, on wet rocks, along streams and springs, on moist gravelly substrates, and in subalpine and alpine grasslands, largely independently of bedrock type. Its wider geographical distribution includes northern regions of Europe, Asia and North America, as well as mountain systems farther south in Europe [38,39,40].

### 4.3. Field Sampling and Morphological Measurements

A total of 338 flowering individuals of *V. biflora* were sampled in 2018 at 34 sites spanning an elevational gradient from 890 to 2115 m a.s.l. (Figure 4). Sampling sites were selected to provide approximately even coverage of the species’ elevational range in the study area, allowing straight-line and curvilinear relationships between morphological traits and elevation to be modelled. Geographic coordinates and elevations of all sampling sites are provided in Table 3. Plant collection was conducted under a permit issued by Tatra National Park (permit no. DBN.503/28/18).

The vegetation in which *V. biflora* occurred changed considerably along the gradient. Up to approximately 1400 m a.s.l., plants were sampled mainly in forest understorey vegetation, with occasional sampling at forest edges. At higher elevations, the species occurred predominantly in open subalpine and alpine vegetation, including grasslands on gravelly and rocky substrates (Table 3).

At each site, 10 flowering individuals bearing a single flower were sampled at peak flowering, except at two sites where nine plants were collected. Because most individuals bore only one flower, irrespective of elevation, sampling was restricted to single-flowered individuals to standardise reproductive biomass with respect to flower number. Sampled individuals were separated by at least 1.5 m to reduce the likelihood of repeatedly sampling the same genet. Only apparently healthy plants without visible symptoms of herbivory, disease, or physical damage were selected.

Floral dimensions were measured immediately after collection in the field, while the flowers retained their natural shape. Corolla length (CorL), corolla width (CorW), and lower petal width (PetalW) were measured with a digital calliper (Powerfix Profi, Neckarsulm, Germany), as illustrated in Figure 5C. Plants were subsequently pressed and dried for further morphological measurements and laboratory analyses. Plant height (PH) was measured from the base of the plant to its highest point. For the largest rosette leaf, petiole length (RLPL) and two perpendicular blade dimensions were measured: the maximum blade diameter (BladeD1) and the diameter perpendicular to BladeD1 (BladeD2) (Figure 5B). Rosette leaf blade area (RLBA) was approximated as the area of an ellipse using Formula (1):

RLBA = π × (BladeD1 × BladeD2)/4,(1)

The same measurements and Formula (1) were applied to the largest stem leaf to determine stem leaf petiole length (SLPL) and stem leaf blade area (SLBA).

For determination of dry mass, plants were separated into stems, leaves and flowers. Plant material was oven-dried for 48 h at 80 °C using a laboratory drying oven with natural air circulation (Pol-Eko-Aparatura SLN 240, Wodzisław Śląski, Poland), following Pérez-Harguindeguy et al. [60]. Dry masses were determined with an analytical balance (Radwag AS 60/220.X2 PLUS, Radom, Poland). The analysed biomass traits comprised plant mass (PM), leaf mass (LM), stem mass (SM) and flower mass (FM). Biomass-allocation traits were expressed as leaf mass fraction (LMF), stem mass fraction (SMF) and flower mass fraction (FMF). Individual-level measurement data and derived traits are available in the Supplementary Data File, whereas details of morphological and biomass trait measurements are provided in Table 4.

### 4.4. Statistical Analyses

All statistical analyses were performed in R version 4.5.1 [61]. Elevational variation in morphological and biomass-allocation traits was analysed using linear mixed-effects models based on plants sampled at 34 sites. Analyses included all available observations; sample size was 338 for all traits except rosette leaf blade area and rosette leaf petiole length, for which measurements were available for 327 individuals. Models were fitted with the *lmer()* function from the lme4 package [62], with elevation included as a continuous fixed effect and sampling site treated as a random effect. *P*-values for fixed effects were obtained using the lmerTest package with Satterthwaite’s approximation [63]. Statistical significance was assessed at *P* < 0.05.

To account for potential non-straight-line responses of traits to elevation, two alternative mixed-effects models were fitted for each trait. The first model with elevation included as a fixed effect representing a straight-line relationship was calculated using Formula (2):

Y = a + b_1_ × elevation (2)

where a represents the intercept and b_1_ the regression coefficient. The second model with additionally included a quadratic term for elevation was calculated using Formula (3):

Y = a + b_1_ × elevation + b_2_ × elevation^2^ (3)

where b_2_ denotes the quadratic regression coefficient.

Comparison of these models allowed assessment of whether trait variation along the elevational gradient was better described by a straight-line or curvilinear relationship. Model fits were compared using likelihood-ratio tests based on chi-squared statistics. Because the models differed in their fixed-effects structure, comparisons were based on maximum-likelihood (ML) fits; models initially fitted using restricted maximum likelihood (REML) were automatically refitted using ML by the *anova()* function [64]. A model-selection *P*-value < 0.05 (Table 2) indicated that inclusion of the quadratic term significantly improved model fit relative to the straight-line model, and the quadratic model was therefore retained. Otherwise, the straight-line model was selected.

## 5. Conclusions

*Viola biflora* exhibited pronounced, trait-specific variation in morphology and aboveground biomass allocation across its broad elevational range in the Tatra Mountains. The prediction that plant stature and vegetative organ dimensions would decrease towards the upper part of the elevational range was broadly supported, although several traits showed non-monotonic relationships with elevation. In particular, plant height and both rosette and stem leaf petiole lengths exhibited pronounced hump-shaped relationships, with maxima at intermediate elevations, consistent with our expectation that traits associated with vertical leaf positioning might respond non-monotonically to elevation. These maxima broadly coincided with the transition from forest understorey to open subalpine vegetation, suggesting that changes in vegetation structure and light environment, together with climatic conditions, may contribute to the observed patterns. In contrast, leaf mass and leaf blade area generally declined towards higher elevations. Flower mass showed no significant elevational trend despite decreases in corolla length and width, whereas flower mass fraction increased towards the upper part of the gradient, largely reflecting a decline in vegetative biomass rather than an increase in absolute flower biomass.

The mechanisms underlying the observed elevational patterns remain uncertain, particularly the relative contributions of climatic conditions and competition for light to the mid-elevation maxima in plant height and petiole length. Future studies incorporating measurements of light availability, surrounding vegetation structure and microclimatic conditions could help clarify these relationships and determine whether similar non-monotonic responses occur in other plant species spanning forest-to-alpine transitions. The absence of a significant elevational trend in flower mass also raises questions about whether reproductive performance is maintained despite declining vegetative biomass at high elevations, which could be investigated through measurements of seed production and germination success. Furthermore, examining the frequency and reproductive success of chasmogamous and cleistogamous flowers along the elevational gradient could provide insight into how reproductive strategies vary across the species’ range. Overall, our findings emphasise the importance of considering both climatic gradients and associated changes in vegetation environments when interpreting intraspecific morphological variation in mountain plants.

## Figures and Tables

**Table 1:** Fitted trait values of *Viola biflora* derived from mixed-effects models and percentage changes along the elevational gradient. Fitted values and percentage changes are presented for traits showing statistically significant relationships with elevation (*P* < 0.05); dashes indicate traits or quantities for which values were not calculated. Percentage changes were calculated from unrounded fitted values.

Trait	Value at 890 m a.s.l.	Local Extremum Value	Elevation of Local Extremum (m a.s.l.)	Value at 2115 m a.s.l.	Change: 890 m to Local Extremum (%)	Change: Local Extremum to 2115 m (%)	Total Change Across Gradient (%)
*Vegetative traits*							
Plant mass (mg)	63.78	70.60	1289	41.20	+10.7	−41.6	−35.4
Leaf mass (mg)	53.98	—	—	38.77	—	—	−28.2
Stem mass (mg)	12.95	15.64	1330	7.02	+20.8	−55.1	−45.8
Plant height (mm)	126.3	155.3	1412	102.3	+22.9	−34.1	−19.0
Rosette leaf blade area (cm^2^)	5.04	5.43	1215	2.45	+7.7	−54.9	−51.4
Stem leaf blade area (cm^2^)	4.63	—	—	2.35	—	—	−49.2
Rosette leaf petiole length (mm)	65.2	85.6	1438	54.4	+31.2	−36.4	−16.6
Stem leaf petiole length (mm)	20.3	35.7	1538	23.4	+75.6	−34.5	+15.1
*Floral traits*							
Flower mass (mg)	—	—	—	—	—	—	—
Corolla length (mm)	16.6	—	—	14.0	—	—	−15.5
Corolla width (mm)	14.4	—	—	11.6	—	—	−19.6
Petal width (mm)	—	—	—	—	—	—	—
*Biomass allocation*							
Leaf mass fraction (%)	—	—	—	—	—	—	—
Stem mass fraction (%)	—	—	—	—	—	—	—
Flower mass fraction (%)	5.2	4.1	1326	7.6	−20.7	+85.8	+47.3

**Table 2:** Summary of linear mixed-effects models relating *Viola biflora* traits to elevation. *P_model selection_*, *p*-value of the likelihood-ratio test comparing straight-line and quadratic models; *P*, *p*-value for the elevation term of the selected model (linear term for straight-line models and quadratic term for quadratic models); *ns*, non-significant at *P* ≥ 0.05.

Trait	*P_model selection_*	Intercept *a*	Coefficient *b*_1_	Coefficient *b*_2_	*P*
*Vegetative traits*					
Plant mass (mg)	0.0260	8.511 × 10^−1^	1.082 × 10^−1^	−4.206 × 10^−5^	0.0350
Leaf mass (mg)	0.0637	6.480 × 10^1^	−1.223 × 10^−2^	—	0.0289
Stem mass (mg)	0.0012	−8.802 × 10^0^	3.676 × 10^−2^	−1.383 × 10^−5^	0.0022
Plant height (mm)	0.0002	−5.532 × 10^1^	2.983 × 10^−1^	−1.057 × 10^−4^	0.0005
Rosette leaf blade area (cm^2^)	0.0178	6.684 × 10^−2^	8.847 × 10^−3^	−3.654 × 10^−6^	0.0247
Stem leaf blade area (cm^2^)	0.1828	6.281 × 10^0^	−1.858 × 10^−3^	—	0.0001
Rosette leaf petiole length (mm)	0.0005	−6.040 × 10^1^	2.031 × 10^−1^	−7.025 × 10^−5^	0.0010
Stem leaf petiole length (mm)	0.0007	−5.122 × 10^1^	1.132 × 10^−1^	−3.681 × 10^−5^	0.0014
*Floral traits*					
Flower mass (mg)	0.8519	2.877 × 10^0^	−1.469 × 10^−4^	—	0.3230 *ns*
Corolla length (mm)	0.1174	1.839 × 10^1^	−2.070 × 10^−3^	—	0.0004
Corolla width (mm)	0.3913	1.644 × 10^1^	−2.274 × 10^−3^	—	0.0001
Petal width (mm)	0.0459	3.642 × 10^0^	3.233 × 10^−3^	−1.194 × 10^−6^	0.0586 *ns*
*Biomass allocation*					
Leaf mass fraction (%)	0.5918	7.421 × 10^1^	−5.476 × 10^−4^	—	0.7340 *ns*
Stem mass fraction (%)	0.0492	6.643 × 10^0^	2.287 × 10^−2^	−8.114 × 10^−6^	0.0624 *ns*
Flower mass fraction (%)	0.0019	1.360 × 10^1^	−1.447 × 10^−2^	5.460 × 10^−6^	0.0033

**Table 3:** Study sites of *Viola biflora* in the Polish Tatra Mountains. Geographic coordinates are given in the WGS 84 coordinate reference system.

Site	Elevation (m a.s.l.)	Elevational Belt	Habitat Type	Latitude (N)	Longitude (E)
1	890	lower montane forest	forest	49.2818889	19.9575555
2	918	lower montane forest	forest	49.2776111	19.9425000
3	960	lower montane forest	forest	49.2781389	19.9811111
4	960	lower montane forest	forest	49.2744167	19.9013611
5	965	lower montane forest	forest	49.2599444	19.8691666
6	995	lower montane forest	forest	49.2613889	19.8730833
7	1015	lower montane forest	forest edge	49.2708056	19.9790833
8	1065	lower montane forest	forest edge	49.2646389	19.9817499
9	1085	lower montane forest	forest	49.2635000	19.9759444
10	1090	lower montane forest	forest	49.2626389	19.9750833
11	1140	lower montane forest	forest	49.2691111	19.8828333
12	1157	lower montane forest	forest	49.2564167	19.9708055
13	1212	upper montane forest	forest edge	49.2528611	19.9695277
14	1265	upper montane forest	forest	49.2531111	19.8813611
15	1290	upper montane forest	forest	49.2510000	19.9567777
16	1300	upper montane forest	forest edge	49.2519722	19.8807500
17	1385	upper montane forest	forest edge	49.2506667	19.8849722
18	1400	upper montane forest	grassland	49.2442778	19.9458888
19	1430	upper montane forest	forest edge	49.2499167	19.8860000
20	1470	upper montane forest	grassland	49.2486111	19.8866666
21	1545	subalpine	grassland	49.2433889	19.9078055
22	1615	subalpine	grassland	49.2465833	19.9298611
23	1640	subalpine	grassland	49.2462500	19.9302777
24	1670	subalpine	grassland	49.2459722	19.9308333
25	1677	subalpine	grassland	49.2296389	20.0002222
26	1740	subalpine	grassland	49.2495833	19.9324166
27	1745	subalpine	grassland	49.2406667	19.8913611
28	1785	subalpine	grassland	49.1996944	20.0559444
29	1877	alpine	grassland	49.2275833	19.9911388
30	1977	alpine	grassland	49.2283333	19.9039722
31	2047	alpine	grassland	49.2343611	19.9164722
32	2052	alpine	grassland	49.2076111	19.7633611
33	2090	alpine	grassland	49.2313056	19.9033055
34	2115	alpine	grassland	49.2318056	19.9096666

**Table 4:** Traits of *Viola biflora* analysed in the study.

Trait	Measurement Details	Measurement Resolution
*Vegetative traits*		
Plant mass (PM)—dry mass of the aboveground plant parts.	Calculated as the sum of leaf, stem and flower mass weighed separately.	0.01 mg
Leaf mass (LM)—dry mass of all plant leaves.	Weighed on an analytical balance after drying for 48 h at 80 °C.	0.01 mg
Stem mass (SM)—dry mass of the plant stem.	As above.	0.01 mg
Plant height (PH)—measured from the base of the plant to its highest point.	The straightened stem was measured with a ruler on pressed, dried specimens.	1 mm
Rosette leaf blade area (RLBA)—area of the blade of the largest rosette leaf.	Calculated as the area of an ellipse using the Equation (1).	—
Stem leaf blade area (SLBA)—area of the blade of the largest stem leaf.	As above.	—
Rosette leaf petiole length (RLPL)—length of the petiole of the largest rosette leaf.	Measured using a digital calliper on pressed, dried specimens.	0.1 mm
Stem leaf petiole length (SLPL)—length of the petiole of the largest stem leaf.	As above.	0.1 mm
*Floral traits*		
Flower mass (FM)—dry mass of a flower.	Weighed on an analytical balance after drying for 48 h at 80 °C.	0.01 mg
Corolla length (CorL)—distance between the apices of the upper petal and the lower petal.	Measured using a digital calliper on a fresh, undeformed flower in the field (see Figure 5C).	0.1 mm
Corolla width (CorW)—distance between the apices of the two lateral petals.	As above (see Figure 5C).	0.1 mm
Petal width (PetalW)—maximum width of the lower petal.	As above.	0.1 mm
*Biomass allocation*		
Leaf mass fraction (LMF)—leaf mass divided by aboveground plant mass (PM), expressed as a percentage.	—	—
Stem mass fraction (SMF)—stem mass divided by aboveground plant mass (PM), expressed as a percentage.	—	—
Flower mass fraction (FMF)—flower mass divided by aboveground plant mass (PM), expressed as a percentage.	—	—

## Data Availability

The individual-level morphological and biomass trait data generated in this study are available in the Supplementary Materials (Supplementary Data File).

## Supplementary Materials

The following supporting information can be downloaded at: https://www.mdpi.com/article/10.3390/plants15213368/s1, Supplementary Data File: Individual-level measurements and derived morphological and biomass traits of *Viola biflora*.

## Abbreviations

The following abbreviation is used in this manuscript:

a.s.l.	above sea level
